# Toxins from cone snails: properties, applications and biotechnological production

**DOI:** 10.1007/s00253-008-1385-6

**Published:** 2008-05-01

**Authors:** Stefan Becker, Heinrich Terlau

**Affiliations:** 1 Department of NMR-based Structural Biology, Max-Planck-Institute for Biophysical Chemistry, Am Faßberg 11, D-37077 Göttingen, Germany; 2 Institute for Experimental and Clinical Pharmacology and Toxicology, Universitätsklinikum Schleswig-Holstein, Campus Lübeck, Ratzeburger Allee 160, 23538 Lübeck, Germany

**Keywords:** Conopeptides

## Abstract

Cone snails are marine predators that use venoms to immobilize their prey. The venoms of these mollusks contain a cocktail of peptides that mainly target different voltage- and ligand-gated ion channels. Typically, conopeptides consist of ten to 30 amino acids but conopeptides with more than 60 amino acids have also been described. Due to their extraordinary pharmacological properties, conopeptides gained increasing interest in recent years. There are several conopeptides used in clinical trials and one peptide has received approval for the treatment of pain. Accordingly, there is an increasing need for the production of these peptides. So far, most individual conopeptides are synthesized using solid phase peptide synthesis. Here, we describe that at least some of these peptides can be obtained using prokaryotic or eukaryotic expression systems. This opens the possibility for biotechnological production of also larger amounts of long chain conopeptides for the use of these peptides in research and medical applications.

## Introduction

**Fig. 1:**
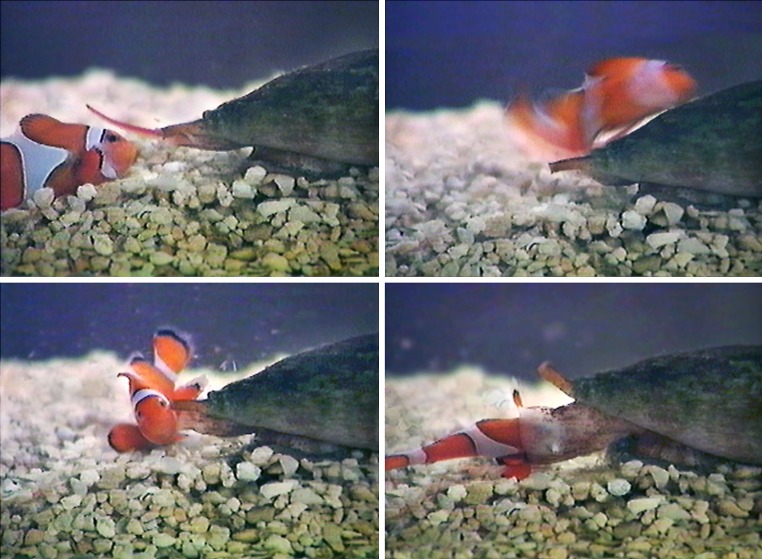
*Conus purpurascens* hunting a clown fish. The snail stings the fish with a harpoon like tooth, which is hollow and barbed and held at the tip of the proboscis. Upon venom injection (*upper right*) the fish is immobilized within less than a few seconds (*lower left*) and engulfed by the snail (*lower right*; originally from Terlau et al. 1996)

Cone snails are mainly known due to the beauty of their shells, which can be found in plenty of the “sea side souvenir shops” all over the world. Commonly not so well recognized is that the biology of these marine snails is very fascinating because these slow animals live as predators. All of the about 500 species known do hunt animals, e.g., other snails, worms, or even fish (see Fig. 1). The immobilization of the prey results from the action of relative complex venoms, which are injected into the victims by using harpoon like teeth. The venom of each species contains up to 200 pharmacologically active components that mainly target different voltage- and ligand-gated ion channels (for overviews see: Olivera et al. 1990; Olivera 2002; Terlau and Olivera 2004). With respect to the venom action on the victim, the different conopeptides can be grouped according to their biological role for the immobilization of prey (for more details see: Olivera 1997). Some conopeptides have been shown to be important for the fast immobilization of the prey (“lightning strike cabal”) whereas others exert their action during later phases of the envenomation, which results in an irreversible block of neuromuscular transmission (“motor cabal”). Because of their special pharmacological properties conopeptides have gained increasing interest in recent years. Moreover, since several conopeptides are currently being tested within clinical trials and the ω-conotoxin MVIIA has obtained an approval as an analgesic drug (Ziconitide, Prialt®) there is an increasing need to produce conopeptides in larger quantities.

In this review, we briefly describe the properties of conopeptides and discuss the current and potential applications of conopeptides in research and clinics. Furthermore, we describe ways of biotechnological production of these peptides, which will help to ease the use of conopeptides within different fields of research and other potential applications.

## Properties of conopeptides

Conopeptides are genetically encoded small proteins that are initially translated as prepropeptide precursors consisting of a signal sequence (the “pre” region) followed by a “pro” region, and at the C-terminal part representing the mature toxin region. The pharmacological active peptide is generated by an enzymatic cleavage of the C-terminal part of the prepropeptide. With respect to their amino acid sequence, conopeptides can be classified in two broad classes: the disulfide-rich peptides and the non-disulfide-rich peptides (Terlau and Olivera 2004).

For the disulfide-rich peptides, several superfamilies have been defined according to the difference in their cysteine connectivity. Within these superfamilies, different peptides may have different targets. Therefore, a given superfamily may consist of several conotoxin families grouped according to their pharmacological properties. The targets of conopeptides are usually voltage-gated and ligand-gated ion channels. For example, within the O-superfamily of disulfide-rich conopeptides that contain six cysteines with an arrangement also known from peptides of different origins (the “inhibitor cystine knot” motif), at least four different families have been identified: the δ-conotoxins, known to inhibit the fast inactivation of voltage-gated Na^+^ channels, the μO-conotoxins that inhibit voltage-gated Na^+^ currents, the κ-conotoxins which interact with voltage-gated K^+^ channels, and the ω-conotoxins known to block voltage-activated Ca^++^ channels. In terms of the immobilization of prey, the peptides from these different families can be involved in the lightning strike cabal or in the motor cabal. For the fish hunting species, *Conus purpurascens*, for example, it has been shown that the δ-conotoxins PVIA and the κ-conotoxin PVIIA are crucial for the lightning strike cabal (Terlau et al. 1996).

One of the most striking features of conopeptides is their pharmacological properties: conopeptides are known to be extraordinarily potent and highly specific. The ω-conotoxin MVIIA, for example, specifically targets N-Type Ca^++^ channels (Ca_v_2.2) with little affinity to other Ca^++^ channel subtypes (Olivera et al. 1987; Hillyard et al. 1992). Since N-type Ca^++^ channels are primarily located in the presynaptic space, the action of ω-conotoxin MVIIA results in blocking synaptic transmission and therefore during envenomation of prey, this peptide is involved in the motor cabal. With respect to the action of the whole venom, the extraordinary specificity of the conopeptides indicates that every single peptide is a “specialist” optimized for a certain target and that only the concerted action of the different peptides present in the venom results in the biological action needed for the achievement of the predatory life of these snails. Due to these pharmacological properties, conopeptides can be ideal tools to study the structure and function of a given target (see below). The finding that peptides with similar cysteine connectivity target different families of ion channels demonstrates that even minor changes in the overall structure of these peptides can result in major pharmacological differences. In fact, these properties might help for the understanding of pharmacological specificity for ion-channel subtypes (i.e., K^+^ channel vs. Na^+^ channel).

Investigations on the venom composition of cone snails suggest that during evolution, every single species of cone snails has developed its own set of conopeptides and it has been estimated that probably more than 50,000 different conopeptides can be found in the venoms of all cone snail species (Terlau and Olivera 2004). Accordingly, conopeptides are extremely variable in their amino acid sequences and the pharmacological properties of only a few of these conopeptides have been characterized in detail so far. The current idea on how the snails have evolved these highly specific peptides within their 50 million years of evolution is that for the cysteine-rich peptides, a hypermutation has occurred for the amino acids in between these cysteines of the mature toxin. Interestingly, in contrast to the mature toxin, the signal sequence of the precursor peptides within a superfamily gene is highly conserved (Olivera 1997, 2006). This high degree of homology can be used for the identification of new peptides from the same family by using PCR techniques.

For the biological function of the cysteine-rich peptides, their disulfide bridges are known to be essential. Furthermore, the disulfide bridges represent a backbone for the structure of these peptides, which are usually relatively stable. The structure of several conopeptides has been solved either by NMR or X-ray analysis.

A striking feature of conopeptides is the presence of a variety of posttranslational modifications, which include hydroxylation of prolines, carboxylation of glutamate, d-amino acids, or sulfated tyrosine (Buczek et al. 2005). There are indications that cone snails express the enzymes important for those modifications in their venom ducts (Stanley et al. 1997; Bandyopadhyay et al. 1998). For γ-carboxylation, it was shown that the enzyme has a recognition signal in the pro region of the precursor. The functional importance of these posttranslational modifications is only partially understood but for the biotechnological production of conopeptides, these modifications introduce some limitations (see below).

## Applications of conopeptides

The extraordinary pharmacological specificity of conopeptides resulted in an intensive use of these peptides for different applications. The ω-conotoxins, for example, are heavily used in neuroscience and also in other areas of research to study the function of Ca^++^-channel subtypes. Accordingly, for the most widely used ω-conotoxin, GVIA, there are about 2,000 research papers published using this peptide as a tool to study these channels.

Due to their specific binding properties, conopeptides can also be valuable tools to obtain structural information about their corresponding target. Especially for transmembrane proteins where no or little structural information is available, the interaction of peptidic toxins can be used to gain structural information of the target protein by using the interaction surface of the peptide as a fingerprint of the interaction surface of the target. Besides conopeptides, also specific toxins from other organisms like scorpions or snakes are used for these studies which have paved the way for obtaining the first structural data on K^+^ channels (Miller 1995; Doyle et al. 1998; MacKinnon et al. 1998). Interestingly, most recent data obtained by solid-state NMR suggest that obviously during the binding of peptide toxins to their target, some conformational changes on both binding partners occur (Lange et al. 2006). These results indicate that at least some conformational dynamics take place during drug–target interactions.

Since voltage-gated and ligand-gated ion channels as the target proteins of conopeptides are involved in a variety of different physiological functions, it becomes immediately clear that certain conopeptides do have the potential to act as lead compounds for new drugs. Accordingly, several conopeptides are currently undergoing clinical trials. Since the application of peptides as drugs is limited it is clear that not always the actual peptide will be the substance of choice for a given indication. But nevertheless, these peptides will help our understanding in how substances do interact with their target and maybe, thereby, opening new fields of pharmaceutical research.

The first example of a conopeptide that is used as a drug is ω-conotoxin MVIIA (Ziconitide, Prialt®) from *Conus magus*, which has been approved for the treatment of intractable pain (Miljanich 2004; Staats et al. 2004; Stix 2005). Interestingly, ω-conotoxin MVIIA is one of the first substances from a marine organism that became a drug used in clinics.

Several other conopeptides are currently being explored as antinociceptive agents. The analgesic effect can be mediated through the interaction with different targets including voltage-activated Ca^++^ channels and Na^+^ channels but also nicotinic acetylcholine receptors, neurotensin receptors, NMDA-type glutamate receptors, and norepinephrine transporter (Olivera 2006). This demonstrates that the specific action of conopeptides can interfere within different signaling pathways involved in pain sensation. In addition, the analgetic action of these peptides helps in the understanding of the molecular mechanisms involved in pain. Only through the discovery that ω-conotoxins are analgetic, for example, did it become clear that N-Type Ca^++^ channels are a potential drug target for severe pain.

**Fig. 2:**
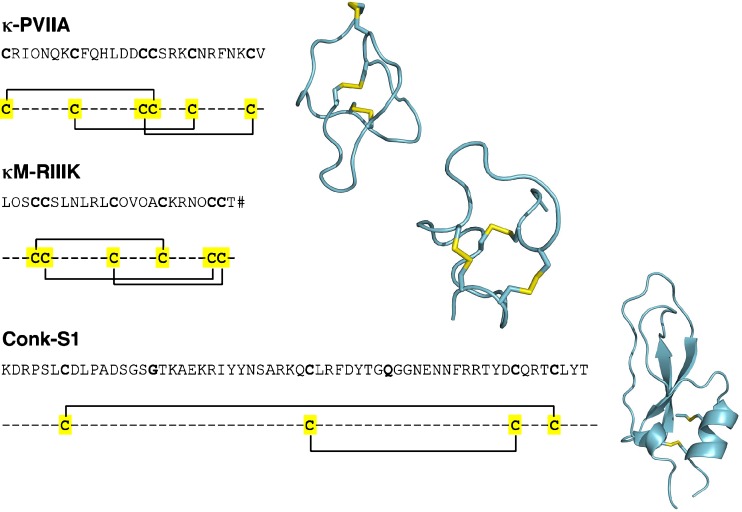
Sequence and structure of K^+^ channel binding conotoxins. Despite a different cysteine backbone and different structures all the three peptides κ-PVIIA (pdb code: 1AV3); κM-RIIIK (*) and Conk-S1 (pdb code: 2CA7) interact with *Shaker* K^+^ channels by occluding the ion channel pore (Shon et al. 1998; Jacobsen et al. 2000; Ferber et al. 2003; Al-Sabi et al. 2004; Bayrhuber et al. 2005; Verdier et al. 2005). *O* 4-hydroxyproline; *Z* pyroglutamate; *#* denotes an amidated C-terminal amino acid. * Structural coordinates obtained from T. Carlomagno

Besides their use as pain killers, conopeptides might also be useful for other clinical indications. For κ-conotoxin PVIIA (see Fig. 2), for example, it has been demonstrated that it reduces the size of the myocardial infarct in an ischemia/reperfusion model in rabbits, rats, and dogs *in vivo* (Zhang et al. 2003; Lubbers et al. 2005). An important feature of this cardioprotective effect is that the reduction was not only observed when κ-PVIIA was applied prior to ischemia, but also if given after the ischemic event and prior to reperfusion, which reflects more the clinical situation.

These examples demonstrate that the biomedical potential of conopeptides is established and that it is very likely that due to the current research on the characterization of their properties, further conopeptides with very interesting pharmacological properties will be discovered.

## Biotechnological production of conopeptides

**Fig. 3:**
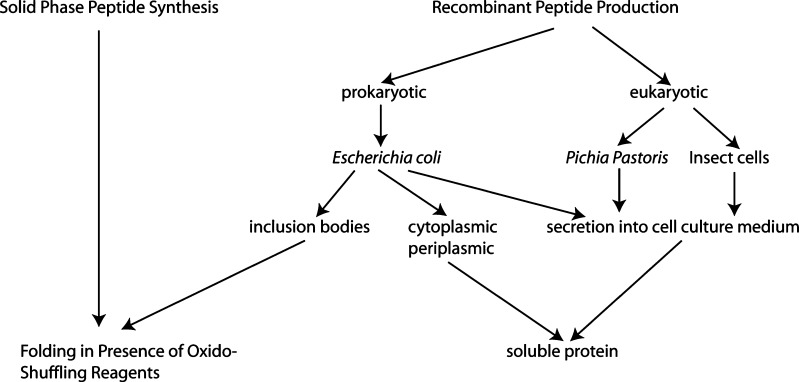
Ways to biotechnological production of disulfide-rich peptides. Solid phase peptide synthesis of disulfide-rich peptides requires subsequent folding of the peptide fragment in the presence of oxygen. Addition of low-molecular-weight thiols in oxidized and reduced form (oxido-shuffling reagents) to the folding solution can help to increase the yield of correctly folded peptide. Recombinant peptide production can result in insoluble protein (inclusion bodies) or soluble protein. From inclusion bodies folding in the presence of oxido-shuffling reagents can be successful

From the natural source, conotoxins can only be obtained in tiny quantities that limit their availability for research and medical applications. To obtain larger amounts of these peptides, two basic approaches are available: chemical synthesis and recombinant production in heterologous expression systems (Fig. 3). Due to the posttranslational modifications of many conotoxins described above (Craig et al. 1999; Buczek et al. 2005), chemical synthesis via solid phase peptide synthesis (SPPS) on a resin support (Merrifield 1963) has been the method of choice to produce conotoxins in large quantities. SPPS using the orthogonal 9-Fluorenylmethyloxycarbonyl (Fmoc)/tertiary-Butyl (tBu) chemistry (Chang and Meienhofer 1978) allows the use of piperidine and trifluoroacetic acid to remove the N-terminal Fmoc and the side chain protection groups, respectively. These low-hazard reagents allow the synthesis of peptides also in typical biology lab environments, making SPPS peptide synthesis available to many life scientists. After removal of the protection groups and cleavage from the resin, the linear peptide needs to be refolded to generate the correct secondary and tertiary structure including the formation of the correct disulfide bond pattern between the cysteine residues present in the polypeptide chain. This oxidation step is usually performed with molecular oxygen (Rudolph and Lilie 1996). The yield of correctly formed disulfide bonds in this step is usually low but can be considerably increased by performing thiol-disulfide exchange reactions with low-molecular-weight thiols that are added in reduced and oxidized form. Typical “oxido-shuffling” reagents are the combinations of reduced and oxidized glutathione, cysteine and cystine, cysteamine and cystamine or di-β-hydroxyethyl disulfide, and 2-mercaptoethanol (Rudolph and Lilie 1996; Bulaj 2005; Lovelace et al. 2006). The thiol–disulfide exchange takes place by nucleophilic attack of the thiolate anion. Therefore, the folding reaction of the disulfide-rich conotoxin peptides is usually performed at mildly alkaline conditions (Moroder et al. 1996; Rudolph and Lilie 1996). SPPS is well functioning for the synthesis up to 30-mer peptides. As most known conotoxins consist of about 10–30 amino acid residues (Terlau and Olivera 2004), SPPS is perfectly suitable for their production. Classical examples for conotoxins synthesized by SPPS are the Ca^++^-channel inhibitor ω-Conotoxin GVIA (Rivier et al. 1987) and µ-Conotoxin GIIIA (Becker et al. 1989), an inhibitor of voltage-dependent Na^+^ channels. Many other conotoxins with posttranslationally modified amino acids have been successfully produced by SPPS in the last 20years.

Chemical ligation of purified peptide fragments can be used to produce longer polypeptides containing up to several hundred amino acids (Dawson and Kent 2000). Using this technique, Conkunitzin-S1 (Conk-S1; see Fig. 2) was recently synthesized (Dy et al. 2006). This conopeptide belongs to a new class of conotoxins that do not contain posttranslational modifications apart from C-terminal amidation and that are structurally strongly related to Kunitz proteins (Bayrhuber et al. 2005; Dy et al. 2006). The expenses for the chemicals and protected amino acids to synthesize such large peptides are quite high. A cost-effective alternative approach to produce such peptides in larger quantities is the use of bacterial and eukaryotic expression systems. The limited posttranslational machinery of these expression systems does not permit the production of peptides with the kind of posttranslational modifications found in many smaller conopeptides (Craig et al. 1999; Buczek et al. 2005). *Escherichia coli* is the best characterized and most widely used bacterial host for the production of recombinant proteins (Baneyx 1999; Pines and Inouye 1999). The reasons are the low costs for culturing *E. coli* and the short culturing times. In general, small peptides are difficult to overexpress directly in *E. coli* since they are either quickly degraded by cellular proteases or they accumulate to form insoluble aggregates, so-called inclusion bodies (Georgiou and Valax 1996). In addition, the cytoplasm of *E. coli* is a reducing environment that prevents the formation of disulfide bonds (Prinz et al. 1997), further increasing the tendency to form inclusion bodies upon overexpression. To achieve at least high expression levels of these small peptides, expressing them in fusion with larger, well-expressing carrier proteins has been the most successful approach so far (Butt et al. 1989; Malakhov et al. 2004). The use of highly soluble carrier proteins like, e.g., maltose-binding protein (Kapust and Waugh 1999), thioredoxin (Lavallie et al. 1993), or glutathione *S*-transferase (Nygren et al. 1994) can even lead to the expression of soluble fusion proteins. Through affinity chromatography targeting such carrier proteins, these soluble fusion proteins can often be easily purified (Nygren et al. 1994; Esposito and Chatterjee 2006; Peti and Page 2007). The additional use of protease-deficient host strains (Maurizi 1992; Gottesman 1996) can help to avoid non-specific proteolytic degradation. From the refolded or soluble fusion protein, the peptide has to be released by chemical or proteolytic cleavage at a pre-engineered cleavage site located in the protein sequence between the carrier protein and the target peptide sequence (Esposito and Chatterjee 2006). Following this approach, the medically important ω-conotoxin MVIIA (Ziconitide) was produced in soluble form as a fusion protein with glutathione *S*-transferase (Xia et al. 2006). Very recently, the newly discovered Conotoxin lt7a was produced in soluble form in fusion with thioredoxin (Pi et al. 2007).

When soluble expression can not be achieved, the protein must be solubilized from the inclusion bodies with denaturing reagents like urea and guanidinium hydrochloride and subsequently refolded by dilution into or by dialysis against a target buffer containing no denaturant (Fahnert et al. 2004). Similar to the formation of disulfide bonds in chemically synthesized peptides (see above), the yield of this refolding step can often be strongly increased by addition of oxido-shuffling reagents (Rudolph and Lilie 1996). In this way, the recombinant conopeptide Conk-S1 has been refolded (Bayrhuber et al. 2006; see also below).

Alternatively, strains with knocked-off oxidoreductases like, e.g., *E. coli* Origami (Novagen) offer a moderately oxidizing environment in their cytoplasm and can be used to increase the fraction of correctly folded disulfide-bonded proteins (Prinz et al. 1997). Although it seems that expression levels tend to be low in such strains (Xiong et al. 2005), this approach has been successfully used for soluble expression of proteins with disulfide bonds (for example, see Lauber et al. 2001; Lehmann et al. 2003; Rabhi-Essafi et al. 2007). But a successful expression of small disulfide-rich peptides like the conotoxins has not been reported so far in such a strain.

Another possibility to obtain soluble fusion proteins of disulfide-rich peptides is the expression into the oxidative environment of the periplasm using a signal peptide sequence in the expression construct. This approach has been successfully used for the periplasmic expression of dendrotoxins and scorpion toxins as fusion proteins with maltose-binding protein (Ducancel et al. 1989; Legros et al. 1997; Legros et al. 2001). More recently, peptides with the inhibitor cystine knot (ICK) structural motif have even been successfully secreted into the culture medium by expressing them as barnase fusion proteins (Schmoldt et al. 2005). Periplasmic expression approaches have not been successfully used for the small disulfide-rich conotoxins. Oxidative folding in the periplasmic space seems to work efficiently only for specific disulfide bridge patterns.

In most fusion protein expression vectors with engineered protease cleavage site, the cloning sites for the peptide coding sequence are located downstream of the coding sequence for the protease recognition site. Therefore, the proteolytic release of peptides from their fusion partner has the potential disadvantage that the cleaved-off peptide may contain additional N-terminal amino acid residues due to cloning. Proteases may also cleave at sites different from the engineered cleavage site and destroy the peptide of interest. In addition, the expenses for proteases can account for a considerable portion of the production costs of recombinant peptides. Chemical cleavage, e.g., by CNBr at methionine residues placed between the fusion partner and the peptide sequence, apart from requiring high lab-safety standards, can also happen at other methionine residues that may be in the peptide.

The production of peptides as fusion proteins with inteins is a way to avoid potential problems with proteolytic or chemical cleavage. Inteins are segments of proteins that are able to excise themselves by a self-catalytic mechanism and rejoin the two flanking parts, the N- and C-exteins, with a peptide bond (Perler and Adam 2000; Saleh and Perler 2006). This splicing reaction is typically initiated by an N to S or N to O acyl shift of Cys1 or Ser1 at the N-terminus of the intein. The resulting (thio)ester is attacked by the first residue of the C-extein, leading via several intermediates to the formation of a new peptide bond between the exteins. For biotechnological purposes, modified inteins have been designed. For example, in the pTWIN vectors (New England Biolabs), the N-terminal cysteine of intein *Ssp*/ DnaB (Wu et al. 1998) has been changed to alanine to block the splicing reaction. This mutant intein is still able to undergo temperature and pH dependent cleavage of the peptide bond between the C-terminus of the intein and the downstream amino acid, thus releasing the C-terminally fused target protein (Mathys et al. 1999; Wood et al. 1999). The design of the multiple cloning site downstream of the *Ssp*/ DnaB coding sequence in the pTWIN vectors includes a *SapI* restriction site that allows the attachment of the peptide sequence immediately downstream of the intein splice site, thus not leaving any additional N-terminal residues in the peptide after the splice reaction. With such an N-terminal intein fusion construct, recombinant Conk-S1 has been produced (Bayrhuber et al. 2006). The *Ssp*/ DnaB–Conk-S1 fusion protein was expressed in insoluble form and was refolded in a buffer containing oxidized and reduced glutathione as oxido-shuffling reagents (Bayrhuber et al. 2006). Cleavage of the fusion protein was achieved by shift to lower pH (from 7.5 to 6.5) and incubation overnight at room temperature. This type of expression construct was also successfully used for the production of another recombinant conkunitzin (unpublished results) and of recombinant Kaliotoxin (Lange et al. 2006), a potassium channel inhibitor from the venom of the scorpion *Leiurus quinquestriatus*.

In summary, the production of disulfide-bonded proteins in *E. coli* frequently includes the refolding of these proteins from inclusion bodies or secretion into the periplasmic space. Both approaches tend to decrease the final yield. An alternative to the production in *E. coli* is the production of disulfide-bonded proteins in eukaryotic expression systems. The methylotrophic yeast, *Pichia pastoris*, provides an intracellular folding environment similar to mammalian cells and secretion into the surrounding medium can be easily accomplished (White et al. 1994). Using this expression system, a number of highly disulfide-bonded proteins have been produced (Kristensen et al. 1999; Cabral et al. 2003). The level of secretion per cell is fairly low in *P. pastoris* (White et al. 1994). Therefore, high-density fermentation was originally a requirement to produce larger amounts of secreted proteins. More recently, the coexpression of protein disulfide isomerase, which probably reduces the retention of disulfide-bonded proteins in the endoplasmic reticulum, has led to enormous improvements of expression yields also in less dense cultures (Vad et al. 2005; Tsai et al. 2006; Huo et al. 2007). Similar to *E. coli*, *P. pastoris* requires no special equipment for handling and the growth media are not more expensive than those for *E. coli.* Thus, production of disulfide-bonded proteins in *P. pastoris* has developed into an attractive alternative to production in *E. coli*. But a successful production of conopeptides has not been reported so far. Insect cells are also used for the production of disulfide-bonded proteins with the *Baculovirus* system (Vogel et al. 2004; Galesi et al. 2007). Stably transfected *Drosophila melanogaster* S2 cells (Galesi et al. 2007) are another insect cell expression system. Recently, psalmotoxin 1, an acid-sensing ion channel inhibitor, was successfully produced in a stable *D. melanogaster* S2 cell line (Escoubas et al. 2003). Due to the high costs of insect cell media, a more wide-spread use of insect cells for the production of highly disulfide-bonded peptides is currently unlikely.

A central consideration of biotechnological protein production is cost-effectiveness. Of all approaches described here, production in *E. coli* is the least expensive because of the very low costs for the culture media and the low investments necessary for culturing these bacteria. Thus, despite of the difficulties encountered, especially with the production of highly disulfide-bonded proteins and peptides like, e.g., the conopeptides, *E. coli* remains the system of choice for their recombinant production. Only the yeast, *P. pastoris*, having the advantage compared to *E. coli* of providing an intracellular folding environment similar to that of mammalian cells, provides an excellent alternative to bacterial expression systems. Other eukaryotic expression systems like, e.g., insect cells or even mammalian cells require prohibitively expensive culture media and expensive cell culture equipment. Regarding the large group of small conotoxins with posttranslational modifications, their production is, at present, still mostly confined to chemical synthesis, e.g., by SPPS. In vitro systems comprising posttranslational modification enzymes to transform recombinant precursors into bioactive peptides have been successfully used (Ozawa et al. 2007). But at present, they offer no higher cost-effectiveness compared to the chemical synthesis and may only be used in the future in cases where refolding of chemically synthesized, highly disulfide-bonded peptides with modified amino acids has not been successful.

Altogether, for the majority of the presently known conopeptides, chemical synthesis is the most important method to produce large amounts of peptide. Recombinant production remains confined to conopeptides without posttranslational modifications. The rapid development of these methods in recent years may allow the biosynthetic production of some modified peptides in the future.
